# Effect of different dentifrices on surface roughness of conventional and additively manufactured resin-based restorative materials

**DOI:** 10.1007/s10266-026-01465-6

**Published:** 2026-06-15

**Authors:** Sema Yazici Akbiyik, Esra Yildirim Manav, Cansu Yikici Çöl

**Affiliations:** 1 Department of Restorative Dentistry, Faculty of Dentistry, Lokman Hekim University, Söğütözü, 2179. Street. No:6, Çankaya, 06530 Ankara, Turkey; 2 Panorama Ankara Oral and Dental Health Polyclinic, Ankara, Turkey

**Keywords:** Surface roughness, Toothbrushing, Resin composite, Additive manufacturing, Three-dimensional printing

## Abstract

This study aimed to evaluate changes in surface roughness after simulated toothbrushing in conventional and additively manufactured resin-based restorative materials using different dentifrices. Four conventional resin composites (flowable, ormocer-based, supra-nano-filled, and bulk-fill) and two additively manufactured restorative resins were included. Specimens were subjected to five brushing conditions: distilled water (control), fluoridated, bioactive, probiotic, and whitening dentifrices (*n* = 10). Toothbrushing was performed for 5,000 cycles under a 200-g load. Surface roughness (Ra) was measured before and after brushing using a contact profilometer, and surface morphology was qualitatively assessed by scanning electron microscopy. Data were analyzed using two-way analysis of variance followed by post hoc tests (α = 0.05). No significant main effect of restorative material (*p* = 0.540) or dentifrice type (*p* = 0.645) was observed; however, a significant material–dentifrice interaction was detected (*p* < 0.001). Pairwise comparisons revealed material-dependent differences in roughness change following brushing. Within the limitations of this in vitro study, post-brushing surface roughness was influenced by the specific combination of restorative material and dentifrice. These findings may be relevant when selecting restorative materials and dentifrices for clinical use.

**Supplementary Information:**

The online version contains supplementary material available at https://doi.org/10.1007/s10266-026-01465-6.

## Introduction

 Resin-based restorative materials are widely used in contemporary dentistry because of their esthetics and adhesive properties. In addition to conventional composite resins such as flowable and bulk-fill materials, additively manufactured resin-based restorative materials have recently been introduced with the development of three-dimensional (3D) printing technologies, offering improved standardization and manufacturing efficiency [1–3].

The clinical performance of these materials is influenced not only by their mechanical properties but also by their surface characteristics. Surface roughness affects plaque retention, discoloration, and interaction with oral tissues, and values exceeding approximately 0.2 μm have been associated with increased bacterial adhesion [4–6]. Therefore, maintaining surface integrity is clinically relevant.

Toothbrushing is a major factor affecting restoration surfaces [7]. Dentifrices differ in their composition and particle systems, and may produce material-dependent surface alterations during daily use [7–9]. Previous studies have generally evaluated either multiple dentifrices on a single restorative material or multiple materials under a single dentifrice. These studies have consistently reported that dentifrices can significantly affect surface roughness, although the magnitude of this effect varies depending on the material composition and the abrasive characteristics of the dentifrice [10–12]. However, patients commonly use one toothpaste in the presence of different restorative materials, making the interaction between material type and dentifrice formulation clinically important [10, 12].

Information regarding the behavior of additively manufactured restorative materials under toothbrushing conditions remains limited, and comparative data with conventional composites within the same experimental design are scarce [12].

Therefore, the aim of this study was to evaluate how simulated toothbrushing with different dentifrice formulations influences surface roughness in conventional and additively manufactured resin-based restorative materials, and whether this effect varies according to material type.

The null hypotheses were that (1) restorative material type would not affect surface roughness change (ΔRa), (2) dentifrice type would not affect surface roughness change (ΔRa), and (3) no interaction would exist between the two factors.

## Materials and methods

### Study design and sample size

This in vitro study was designed to evaluate the surface roughness changes of different resin-based restorative materials following simulated toothbrushing with various dentifrices.

The experimental workflow consisted of three main stages: specimen preparation, simulated toothbrushing under different dentifrice conditions, and surface roughness measurements performed before and after brushing. This stepwise design was adopted to ensure a clear and standardized experimental procedure.

Sample size was calculated using G*Power software (version 3.1.9.7; Heinrich Heine University Düsseldorf, Germany). Based on a previous study [13], a medium effect size (f = 0.50), α = 0.05, and power (1−β = 0.80) were used, resulting in a minimum of eight specimens per group. To increase reliability and account for possible sample loss, ten specimens were included in each group.

A total of 30 experimental groups were created by combining six different restorative materials with five dentifrice conditions (6 × 5). For each group, ten disc-shaped specimens were prepared, resulting in a total of 300 specimens.

Six resin-based restorative materials were evaluated. The conventional materials included:

G: GC G-ænial Universal injectable (GC, Tokyo, Japan), a flowable composite resin.

A: Admira Fusion Flow (VOCO, Cuxhaven, Germany), an ormocer-based flowable composite resin.

OM: Omnichroma (Tokuyama Dental, Tokyo, Japan), a condensable composite resin.

E: Estelite Bulk Flow (Tokuyama Dental, Tokyo, Japan), a bulk-fill flowable composite resin.

The additively manufactured resin-based materials:

D: Saremco Crowntec (Saremco Dental, Rebstein, Switzerland).

B: Bego VarseoSmile Crown Plus (BEGO, Bremen, Germany).

Detailed compositional characteristics of the materials are presented in Table 1.

**Table 1:** Resin-based restorative materials used in the study and their compositions

Material	Lot	Organic matrix	Filler content / structural characteristics
GC G-ænial universal injectable (GC Tokyo, Japan)	240823 A	Methacrylate-based resin matrix	Barium glass and silica fillers (~46 vol%)
Admira fusion flow (VOCO, Cuxhaven, Germany)	2423412	Ormocer-based resin matrix	Barium-aluminium-borosilicate glass inorganic fillers (~74 wt%)
Omnichroma (Tokuyama Dental, Tokyo, Japan)	103M45	UDMA-based dimethacrylate TEGDMA,	Spherical silica–zirconia nano fillers (~68 vol%, 79 wt%)
Estelite bulk flow (Tokuyama Dental, Tokyo, Japan)	093E13	Bis-GMA/Bis MPEPP/TEGDMA/UDMA dimethacrylate resin matrix	Supra-nano Spherical SiO₂–ZrO₂ nano-fillers (~70 wt%)
Saremco Crowntec (Saremco Dental, Rebstein, Switzerland)	F298	UDMA- Bis-GMA- TEGDMA	Inorganic glass fillers (~50–60 wt%)
Bego VarseoSmile Crown Plus (BEGO, Germany)	602,021	UDMA/ TEGDMA Aliphatic dimethacrylates-	Microfine ceramic fillers (~40–50 wt%)

*Bis-GMA* bisphenol-A glycidyl methacrylate, *Bis-MPEPP* bisphenol-A ethoxylated dimethacrylate, *UDMA* urethane dimethacrylate, *TEGDMA* triethylene glycol dimethacrylate, *Ormocer* organically modified ceramic, *Dimethacrylate* cross-linking methacrylate resin monomer system, *wt%* weight%, *vol%* volume percentage, *SiO₂* silicon dioxide, *ZrO₂* zirconium dioxide

Five different toothbrushing conditions were defined based on dentifrice type:

Group 0 (control): toothbrushing with distilled water.

Group 1: fluoridated dentifrice.

Group 2: bioactive dentifrice (containing hydroxyapatite and whitening agents).

Group 3: probiotic dentifrice.

Group 4: whitening dentifrice.

The composition of dentifrices are given in Table 2. Dentifrice abrasivity parameters (RDA values) were not evaluated, as the primary aim of the study was to assess the resulting surface roughness after brushing rather than the intrinsic abrasivity of the dentifrices. Similar approaches have been adopted in previous studies focusing on surface outcomes following toothbrushing simulations [7, 13].

**Table 2:** Dentifrices used in the study and their detailed compositions

Group	Toothpaste/manufacturer	Composition
Group 0 control	Control (Toothbrushing without dentifrice)	Mechanical brushing only with distilled water; no chemical agents
Group 1 fluoridated dentifrice	Colgate total active; colgate-palmolive, New York, NY, USA	Stannous flouride, sodium fluoride, sorbitol, aqua, hydrated silica, glycerin, aroma, sodium lauryl sulfate, tetrasodium pyrophosphate, cellulose gum, potassium nitrate, xanthan gum, sodium saccharin, cocamidopropyl betaine, Cl 77891
Group 2 bioactive dentifrice	Ultracomplex bio-active toothpaste; SPLAT, Moscow, Russia	Aqua, hydrogenated starch hydrolysate hydrated silica, PEG-8, sodium coco-sulfate, cellulose gum, aroma, sodium bicarbonate, potassium nitrate, calcium lactate, zinc citrate, sodium methylparaben, PVP, calcium hydroxyapatite, sodium saccharin, bisabolol, papain, mannitol, farnesol, Cl 77891, fluoride free
Group 3 probiotic dentifrice	Probident active probiotic toothpaste/grape seed extract; probident, Istanbul, Turkiye	Aqua, glycerin, hydrated silica, calcium carbonate, xylitol, aroma, xanthan gum, rebaudioside A, phenylpropanol, cocamidopropyl betaine, caprylyl glycol, vitis vinifera seed extract, boswellia serrata extract, tetrahydrocurcumin, menthol, maltodextrin, bacillus, phenoxyethanol, benzoic acid, dehydoacetic acid, limonene
Group 4 whitening dentifrice	Opalescence whitening toothpaste; ultradent products, South Jordan, UT, USA	Glycerin, water(aqua), silica, sorbitol, xylitol, flavor (aroma), poloxamer 407, sodium lauryl sulfate, carbomer, sodium benzoate, sodium hydroxide, sucralose, xanthan gum, FD&C blue #1 (CI 42090), FD&C yellow #5 (CI 19140)

### Specimen preparation

#### Composite resin specimens

Disc-shaped specimens (8 mm in diameter and 2 mm in thickness) were prepared using Teflon molds. Mylar strips were placed on the top and bottom surfaces of the molds, and a glass slide was applied with gentle pressure to obtain a smooth initial surface.

Polymerization was performed using an LED light-curing unit (Woodpecker Led-B, China) according to the manufacturers’ instructions. After curing, the specimens were removed from the molds.

#### Additively manufactured resin-based restorative materials specimens

Additively manufactured specimens were produced using a DLP-based 3D printer (T-rox, Tolard, Türkiye). All specimens were printed in a standardized build orientation. Following printing, the specimens were cleaned in an ultrasonic bath containing 96% isopropyl alcohol for 5 min to remove uncured resin residues and then air-dried. Post-curing was performed using a LED-based unit (Otoflash G171, NK-Optik, Germany) under a nitrogen atmosphere, with two consecutive cycles of 1500 light pulses each.

All specimens, including both conventionally fabricated and additively manufactured materials, were subjected to a standardized polishing protocol prior to testing to minimize surface irregularities and ensure comparable initial surface conditions across all groups. The surfaces were polished using aluminum oxide discs (Sof-Lex, 3 M, USA) applied respectively (coarse, medium, fine, and superfine) for 10 s each, resulting in a uniform surface finish [13]. After polishing, the specimens were stored in distilled water at 37 °C for 24 h.

### Toothbrushing simulation protocol

All procedures were performed by a single calibrated operator blinded to group allocation. Specimens were mounted in a mechanical toothbrushing simulator (Toothbrush Simulator BS12, Lua, Istanbul, Türkiye) with the polished surface oriented upward. Brushing was performed at a frequency of 5 Hz under a constant load of 200 g (1.96 N). A soft toothbrush (Colgate Classic Toothbrush, Colgate-Palmolive, USA) was used, with the brush head positioned parallel to the specimen surface [7].

A slurry was prepared by mixing dentifrice and distilled water in a 1:3 ratio by weight [7]. Toothbrushing was simulated using 5,000 cycles, which is commonly considered to represent approximately six months of clinical brushing in laboratory studies [7, 14]. After brushing, specimens were rinsed with distilled water and stored again at 37 °C for 24 h prior to final measurements. To ensure consistent positioning during brushing and measurement, a reference mark was placed on the non-tested (bottom) surface near the edge of each specimen using a diamond bur, without affecting the polished test surface. This marking enabled accurate repositioning of the specimens in the toothbrushing device and ensured a consistent orientation perpendicular to the brushing direction during measurements.

### Surface roughness measurement

Surface roughness was measured before and after brushing using a contact profilometer (Perthometer; Mahr, Germany). The device was calibrated prior to measurements.

Each specimen was positioned perpendicular to the stylus, and three measurements were performed at different locations using a cut-off value of 0.25 mm and a tracing length of 5.5 mm. The mean of the three readings was recorded as the average roughness (Ra, µm).

Surface roughness was evaluated using Ra values, as this parameter is widely used in dental materials research and allows for comparison with previous studies [7, 13]. 

### Scanning electron microscopy (SEM)

For qualitative analysis of surface topography, one test specimen was randomly selected from each group, and the central area of each specimen was examined using a scanning electron microscope (SEM; Zeiss Ultra Plus, Germany) at ×1000 magnification. Prior to SEM examination, the specimens were sputter-coated with a gold–palladium (Au/Pd) alloy using a coating device (Quorum Q150R ES, UK) to enhance surface conductivity and prevent charging.

### Statistical analysis

Statistical analyses were performed using SPSS (version 26.0; IBM, Armonk, NY, USA). The primary outcome variable was the change in surface roughness (ΔRa), calculated as the difference between post-brushing and baseline values.

Normality of data distribution was assessed using the Shapiro–Wilk test, and homogeneity of variances was evaluated using Levene’s test. The effects of restorative material and dentifrice type on ΔRa were analyzed using two-way ANOVA. When significant effects were detected, pairwise comparisons were performed using Tukey post hoc tests.

Within-group comparisons of baseline and post-brushing roughness values were performed using paired t-tests. The level of significance was set at α = 0.05.

## Results

Surface roughness change was calculated as ΔRa (Ra_after − Ra_before). Two-way ANOVA showed no significant main effect of restorative material (*p* = 0.540) or dentifrice type (*p* = 0.645), whereas a significant material–dentifrice interaction was detected (*p* < 0.001).

Within individual materials, significant differences among dentifrices were observed (Table 3).

**Table 3:** Roughness change (ΔRa, µm) according to dentifrice type within each restorative material

Material	0	1	2	3	4
A	0.012±0.049^a^	-0.021±0.023^a^	0.060±0.081^b^	0.047±0.064^b^	0.062±0.031^b^
G	0.026±0.022^b^	0.027±0.040^b^	0.036±0.035^b^	0.007±0.027^ab^	-0.017±0.024^a^
OM	-0.057±0.072^a^	0.080±0.064^b^	0.019±0.022^ab^	-0.023±0.056^a^	0.080±0.098^b^
E	0.004±0.020^ab^	0.056±0.077^ab^	0.015±0.035^ab^	0.066±0.069^b^	0.001±0.016^a^
D	0.099±0.191^b^	-0.019±0.049^ab^	-0.052±0.027^a^	-0.029±0.047^ab^	0.036±0.122^ab^
B	0.054±0.134^a^	0.125±0.347^a^	0.002±0.150^a^	0.052±0.123^a^	-0.015±0.154^a^

Different superscript letters within the same row indicate statistically significant differences (Tukey test, *p* < 0.05)

Material codes: A = Admira Fusion Flow; G = G-ænial Universal Injectable; OM = Omnichroma; E = Estelite Bulk Flow; D = Saremco Crowntec; B = Bego VarseoSmile Crown Plus

Dentifrice codes: 0 = control (water), 1 = fluoridated, 2 = bioactive, 3 = probiotic, 4 = whitening

Omnichroma showed higher ΔRa values with fluoridated and whitening dentifrices compared with the control condition. Saremco Crowntec showed lower ΔRa with the bioactive dentifrice than with the control. In G-ænial Universal Injectable, the whitening dentifrice resulted in lower ΔRa than the other dentifrices. Estelite Bulk Flow showed higher ΔRa with the probiotic dentifrice than with the whitening dentifrice, whereas Bego VarseoSmile Crown Plus showed no significant differences among dentifrices.

Comparisons among restorative materials within each dentifrice condition showed significant differences under control, bioactive, and probiotic conditions, but not under fluoridated or whitening dentifrices (Table 4). Under the control condition, Saremco Crowntec showed higher ΔRa than Omnichroma. Under the bioactive dentifrice, Admira Fusion Flow showed higher ΔRa values than Saremco Crowntec.

**Table 4:** Roughness change (ΔRa, µm) according to restorative material within each dentifrice condition

Dentifrice	A	G	OM	E	D
0	0.012±0.049^ab^	0.026±0.022^ab^	-0.057±0.072^a^	0.004±0.020^ab^	0.099±0.191^b^
1	-0.021±0.023^a^	0.027±0.040^a^	0.080±0.064^a^	0.056±0.077^a^	-0.019±0.049^a^
2	0.060±0.081^b^	0.036±0.035^ab^	0.019±0.022^ab^	0.015±0.035^ab^	-0.052±0.027^a^
3	0.047±0.064^ab^	0.007±0.027^ab^	-0.023±0.056^ab^	0.066±0.069^b^	-0.029±0.047^a^
4	0.062±0.031^ab^	-0.017±0.024^a^	0.080±0.098^b^	0.001±0.016^ab^	0.036±0.122^ab^

Different superscript letters within the same row indicate statistically significant differences (Tukey test, *p* < 0.05)

Material codes: De: Dentifrice; A = Admira Fusion Flow; G = G-ænial Universal Injectable; OM = Omnichroma; E = Estelite Bulk Flow; D = Saremco Crowntec; B = Bego VarseoSmile Crown Plus

Dentifrice codes: 0 = control (water), 1 = fluoridated, 2 = bioactive, 3 = probiotic, 4 = whitening

Within-group comparisons demonstrated significant differences between baseline and post-brushing Ra values in several material–dentifrice combinations (Table 5).

**Table 5:** Surface roughness (Ra, µm) before and after toothbrushing and roughness change (ΔRa) for each material–dentifrice combination

Material	Dentifrice	Baseline Ra (µm)	Post-brushing Ra (µm)	ΔRa (µm)	*p*-value
A	0	0.249±0.028	0.261±0.033	0.012±0.049	0.456
A	1	0.202±0.017	0.181±0.020	-0.021±0.023	0.018
A	2	0.179±0.065	0.238±0.020	0.060±0.081	0.045
A	3	0.188±0.060	0.235±0.018	0.047±0.064	0.045
A	4	0.253±0.034	0.315±0.014	0.062±0.031	<0.001
B	0	0.355±0.137	0.409±0.089	0.054±0.134	0.236
B	1	0.538±0.180	0.663±0.230	0.125±0.347	0.283
B	2	0.400±0.115	0.402±0.100	0.002±0.150	0.972
B	3	0.497±0.115	0.549±0.052	0.052±0.123	0.215
B	4	0.345±0.071	0.331±0.145	-0.015±0.154	0.768
D	0	0.311±0.133	0.409±0.102	0.099±0.191	0.136
D	1	0.211±0.029	0.192±0.046	-0.019±0.049	0.258
D	2	0.189±0.026	0.138±0.024	-0.052±0.027	<0.001
D	3	0.194±0.050	0.164±0.014	-0.029±0.047	0.082
D	4	0.295±0.100	0.331±0.066	0.036±0.122	0.379
E	0	0.116±0.006	0.121±0.018	0.004±0.020	0.499
E	1	0.216±0.032	0.271±0.047	0.056±0.077	0.049
E	2	0.098±0.007	0.114±0.030	0.015±0.035	0.201
E	3	0.104±0.015	0.170±0.062	0.066±0.069	0.015
E	4	0.113±0.014	0.114±0.005	0.001±0.016	0.878
G	0	0.108±0.022	0.134±0.013	0.026±0.022	0.005
G	1	0.071±0.025	0.098±0.032	0.027±0.040	0.065
G	2	0.095±0.011	0.131±0.041	0.036±0.035	0.010
G	3	0.121±0.013	0.128±0.033	0.007±0.027	0.438
G	4	0.122±0.015	0.105±0.027	-0.017±0.024	0.049
OM	0	0.221±0.038	0.163±0.065	-0.057±0.072	0.033
OM	1	0.095±0.021	0.175±0.065	0.080±0.064	0.003
OM	2	0.112±0.007	0.131±0.019	0.019±0.022	0.025
OM	3	0.171±0.057	0.148±0.016	-0.023±0.056	0.223
OM	4	0.149±0.031	0.229±0.089	0.080±0.098	0.029

Values are presented as mean ± standard deviation

ΔRa was calculated as Ra_after − Ra_before. Positive values indicate roughness increase and negative values indicate surface smoothing

P values refer to paired comparisons within each group

Material and dentifrice codes are defined in Tables 3 and 4

### SEM observations

**Fig. 1:**
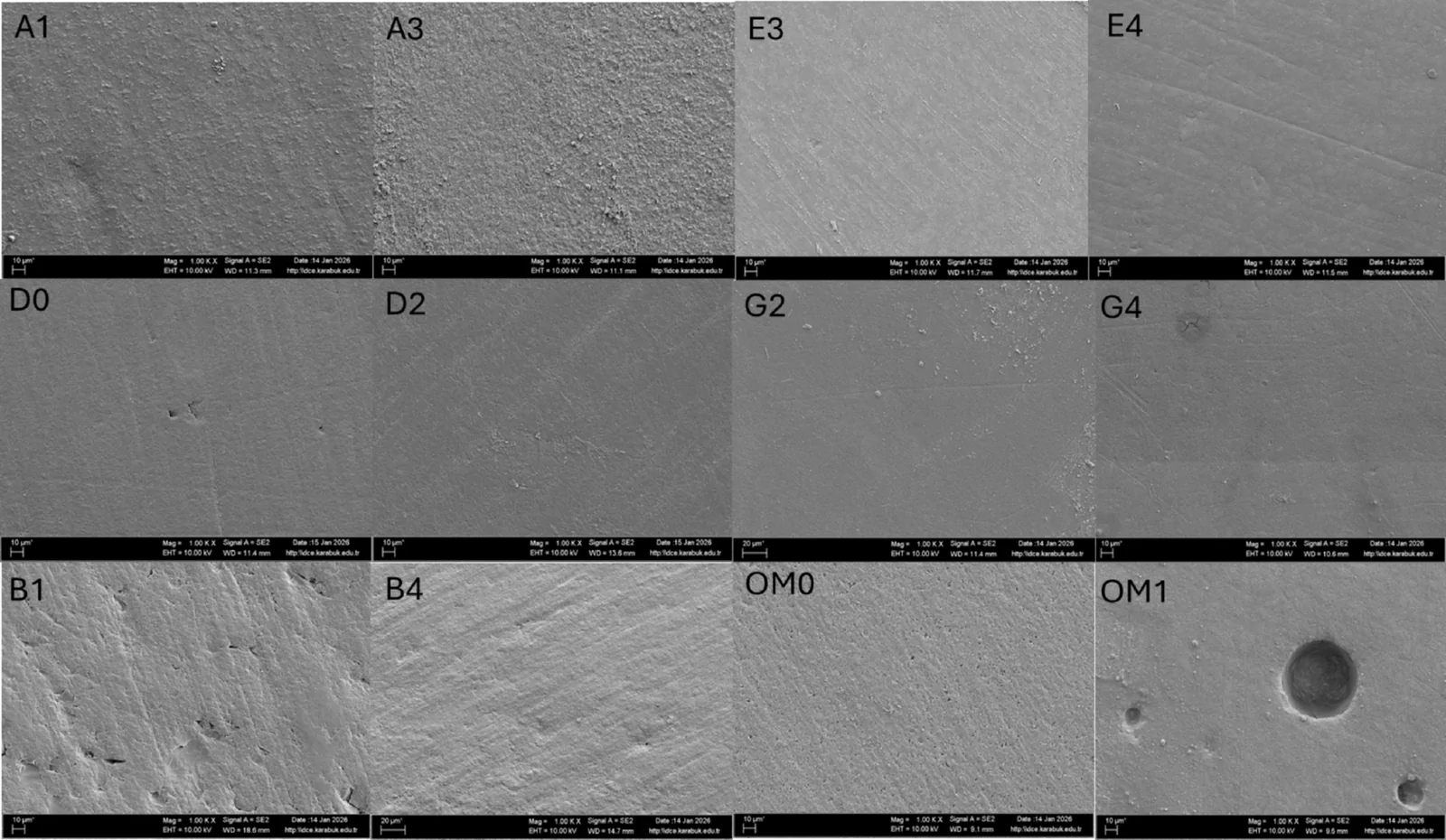
Representative scanning electron microscopy images of the tested restorative materials after simulated toothbrushing (magnification ×1000). Materials: Admira Fusion Flow (A), Omnichroma (OM), Estelite Bulk Flow (E), G-ænial Universal Injectable (G), Saremco Crowntec (D), and Bego VarseoSmile Crown Plus (B). Brushing conditions: 0 = distilled water (control), 1 = fluoridated dentifrice, 2 = bioactive dentifrice, 3 = probiotic dentifrice, and 4 = whitening dentifrice

Representative SEM images are shown in Fig. 1, and all SEM images for 30 groups are provided in the supplementary material (Figure S1).

## Discussion

Surface roughness is an important property influencing the clinical performance of restorative materials [15]. The present study demonstrated that surface roughness changes following toothbrushing are not solely dependent on restorative material or dentifrice type, but rather on their interaction. This finding highlights the importance of evaluating these factors together rather than in isolation, as commonly done in previous studies. Accordingly, the hypotheses related to the main effects were not supported, whereas the hypothesis of no interaction was rejected.

These findings further support that the effect of dentifrices is material-dependent and cannot be generalized across different restorative materials. For instance, Omnichroma showed increased surface roughness with fluoridated and whitening dentifrices compared with the control, whereas Saremco Crowntec exhibited reduced roughness with the bioactive dentifrice. In contrast, Bego VarseoSmile Crown Plus showed no significant differences among dentifrice conditions, indicating more stable surface behavior.

The presence of a significant interaction between restorative material and dentifrice suggests that surface roughness changes cannot be attributed to a single factor alone. Rather, the observed effects appear to result from the combined influence of material microstructure and dentifrice characteristics, including abrasive particle properties and composition. This interaction may be explained by the way abrasive particles affect the material structure, leading to matrix wear, exposure of filler particles, or even their dislodgement depending on the material composition [7, 13, 15].

A toothbrushing machine was used to simulate clinical conditions, as commonly reported in similar studies [7, 16, 17]. Due to the wide variability in brushing habits among individuals, it is difficult to establish a direct relationship between the number of brushing cycles and the actual brushing time [16]. However, based on previous studies examining the interaction between toothpastes and composite resins, it was assumed that the 5,000 brushing cycles applied in the present study were considered to correspond to approximately six months of toothpaste use [7, 15]. Since toothbrushing is mostly performed manually, the applied brushing force can vary from person to person. According to ISO standards, a load between 0.5 and 2.5 N is recommended for toothbrushing simulations [18]. In the present study, a constant load of 200 g (1.96 N) was applied during brushing to ensure standardization. For surface roughness measurements, a contact profilometer commonly used for the quantitative evaluation of roughness in dental materials and providing Ra (average surface roughness) values was preferred [7]. Although Ra does not fully represent the complexity of surface topography, it remains the most widely used parameter in dental materials research, allowing comparison with previous studies [7, 15].

The present findings suggest that the change in surface roughness after brushing was influenced not only by the dentifrice but also by the intrinsic structure of the restorative material. Differences in filler content, particle morphology, resin matrix composition, and manufacturing technique are known to affect resistance to mechanical wear [19], which may explain the varied responses among the tested materials. In addition, the interaction between the resin matrix and abrasive particles may lead to selective wear of the softer matrix phase, exposing filler particles and increasing surface irregularities [7, 20]. Therefore, the observed changes are likely the result of both mechanical abrasion and material-dependent structural properties rather than a single isolated factor.

Admira Fusion Flow, an ormocer-based nanohybrid composite, has a matrix structure that differs from conventional dimethacrylate-based resins. This inorganic–organic hybrid network is often associated with reduced polymerization shrinkage and favorable biocompatibility. However, previous studies have suggested that differences at the matrix–filler interface, particularly when combined with relatively larger filler particles, may lead to earlier wear of the resin matrix during brushing, resulting in filler particle exposure or dislodgement [20]. Accordingly, although ormocer-based materials present several advantages, they may not maintain surface smoothness as effectively as nanofilled systems under mechanical wear conditions [20]. In the present study, the behavior of Admira Fusion Flow varied depending on the dentifrice used, which may be related to its distinct matrix–filler interaction. Compared with conventional dimethacrylate-based composites, this material may respond differently to abrasive challenges due to its hybrid network structure, potentially leading to differences in surface roughness changes.

G-ænial Universal Injectable contains uniformly distributed nano-sized fillers that are generally considered beneficial for maintaining surface smoothness [12]. However, its relatively higher organic matrix content may allow preferential wear of the polymer phase during brushing, exposing harder filler particles and producing surface irregularities [7]. In the present study, the variation in roughness depending on dentifrice type suggests that this material’s surface behavior is influenced by its matrix composition. These findings also indicate that resistance to brushing is not uniform even among nanofilled composites and may vary according to differences in composition and microstructure.

Omnichroma, characterized by supra-nano spherical fillers and high filler loading, would be expected to maintain surface stability [21, 22]. Nevertheless, the present study demonstrated that its surface response varied depending on the dentifrice used. This finding suggests that the effect of dentifrices on surface behavior may not be determined solely by filler morphology [12, 22]. When compared with other filler systems, such as nanofilled or hybrid composites, materials with spherical filler particles may still exhibit different responses to abrasive challenges depending on factors such as filler–matrix bonding and particle distribution [8, 23]. Therefore, filler shape alone may not be sufficient to predict surface stability under toothbrushing conditions.

Estelite Bulk Fill Flow, which also contains spherical supra-nano fillers, exhibited roughness changes in certain dentifrice conditions. Although spherical fillers are associated with improved polish retention [12, 22], surface alteration in flowable bulk-fill materials have been related to filler–matrix bonding characteristics rather than viscosity alone [8, 23]. When compared with Omnichroma, which also contains supra-nano spherical fillers, the differences in surface behavior observed in the present study suggest that factors beyond filler morphology such as filler loading, matrix composition, and the quality of filler–matrix interaction may play a more important role in determining resistance to abrasive challenges.

Additively manufactured resin-based materials generally showed more stable behavior compared with conventional composites. Their relatively homogeneous structure and controlled layer-by-layer polymerization may increase resistance to brushing wear. In particular, Bego VarseoSmile Crown Plus showed similar roughness values regardless of dentifrice type. However, long-term brushing simulations indicate that surface degradation may still occur over time [24].

Each material responded differently to the tested dentifrices, and no formulation consistently produced the greatest roughness change. These findings further support the idea that the effect of dentifrices cannot be generalized based solely on their category or marketed function [21, 22]. Instead, their impact appears to depend on the interaction between the formulation of the dentifrice and the structural characteristics of the restorative material.

Under the control condition, brushing with distilled water produced minimal changes in most materials, indicating a limited effect of mechanical action alone. However, the response was not uniform; even without dentifrice, Saremco Crowntec showed a greater roughness increase than Omnichroma, suggesting that intrinsic material properties influence the response to brushing [24]. The greater changes observed in dentifrice groups indicate that the presence of abrasive components in dentifrices contributes to surface alteration, whereas brushing without dentifrice resulted in comparatively minor changes [25]. However, this finding does not suggest that dentifrices should be avoided; rather, it highlights that their formulation may influence the extent of surface changes in restorative materials.

Brushing with fluoridated dentifrice produced variable surface responses depending on the material, although no consistent differences among materials were detected. Since fluoride itself is not abrasive, the observed alterations are likely related to incorporated abrasives [7]. In some materials brushing increased roughness, whereas in others a slight smoothing tendency was observed, suggesting removal of superficial irregularities by fine particles [12, 15]. In the present study, this effect was not consistently observed across all materials, indicating that dentifrice abrasives may produce either smoothing or roughening depending on the material characteristics.

Whitening dentifrices contain abrasive particles with different morphology and hardness [7, 12, 13]. The conventional whitening dentifrice did not produce significant differences among restorative materials, indicating that its effect was not material-selective under the tested conditions. In contrast, the hydroxyapatite-containing whitening dentifrice produced a more pronounced response in certain materials, such as Admira Fusion Flow, suggesting that the interaction between the abrasive system and material microstructure plays a more important role than the presence of hydroxyapatite alone. Although hydroxyapatite may act as a biomimetic component, previous studies have reported that surface alterations in whitening dentifrices are mainly governed by the overall abrasive formulation rather than the active ingredient itself [13, 26].

Brushing with probiotic dentifrice also produced material-dependent surface alterations. Some materials exhibited noticeable roughness changes while others remained stable. Although studies evaluating probiotic-containing dentifrices on restorative materials are limited, previous reports have suggested that probiotic solutions may not significantly affect surface roughness of restorative materials [27]. Conversely, other studies have demonstrated increased roughness on enamel surfaces and on certain restorative substrates following exposure to probiotic formulations [28, 29]. These findings indicate that the effect of probiotic products may vary according to their abrasive characteristics and their interaction with the material microstructure rather than their probiotic content alone.

Relative dentin abrasivity (RDA) is commonly used to estimate the abrasive potential of dentifrices during toothbrushing. Dentifrices are generally classified as having low, medium, or high abrasivity based on their RDA values; however, these categories provide only a general indication of their abrasive behavior. In practice, the effect of a dentifrice on restorative materials depends not only on its RDA value but also on factors such as the size, shape, hardness, and concentration of abrasive particles [30]. In the present study, RDA values and abrasive particle characteristics were not evaluated. Therefore, the interpretation of differences among dentifrices is limited, and the observed variations in surface roughness cannot be attributed solely to abrasivity. Instead, these findings likely reflect the combined influence of multiple formulation-related factors. Although RDA values were not available, the dentifrices used in this study differ in their abrasive systems (e.g., hydrated silica, calcium carbonate), which may have contributed to the observed material-dependent surface responses. As these factors are known to influence surface roughness, their absence should be considered when interpreting the observed differences among dentifrices [30]. Consequently, comparisons among dentifrices in this study should be interpreted with caution, and the findings should not be considered as definitive evidence of differences in abrasive potential.

SEM analysis revealed distinct differences in surface morphology among the tested groups. The SEM observations were generally consistent with the profilometric findings, supporting the quantitative results. Specimens exposed to dentifrices associated with higher ΔRa values exhibited more pronounced surface irregularities, including filler particle exposure and matrix degradation, whereas groups with lower roughness values showed relatively smoother and more homogeneous surfaces.

For example, Omnichroma specimens brushed with fluoridated and whitening dentifrices demonstrated more evident surface irregularities, consistent with their increased roughness values. In contrast, Bego VarseoSmile Crown Plus showed minimal morphological changes across dentifrice conditions, reflecting its relatively stable surface behavior. Overall, the SEM findings qualitatively supported the profilometric results, indicating that surface changes were closely related to the material–dentifrice interaction.

From a clinical perspective, these findings suggest that the long-term surface behavior of restorative materials may vary depending on the dentifrice used. Since increased surface roughness is associated with plaque accumulation, discoloration, and reduced restoration longevity, the interaction between restorative materials and dentifrices should be considered in clinical decision-making. In addition, the final surface condition may be more clinically relevant than the magnitude of roughness change. It has been reported that a surface roughness threshold of approximately 0.2 μm facilitates bacterial adhesion and biofilm accumulation [4–6]. In the present study, several material–dentifrice combinations exceeded this threshold after brushing, which may increase the risk of plaque retention and compromise the long-term performance of restorations. However, these findings should not be directly translated into clinical recommendations, as the study was conducted under in vitro conditions. Nevertheless, they highlight the importance of individualized material selection and patient-specific oral hygiene practices.

A strength of the present study is the evaluation of multiple restorative materials and dentifrices within the same experimental design. Unlike many previous studies that focused on a single material or dentifrice, this study provides a more comprehensive understanding of how different material–dentifrice combinations behave under standardized toothbrushing conditions. This approach enhances the clinical relevance of the findings.

Overall, the results of this study emphasize that the surface behavior of restorative materials under toothbrushing conditions is complex and primarily governed by the interaction between material properties and dentifrice composition, rather than by a single factor alone.

This study has several limitations. The experimental design did not include saliva, thermal cycling, pH fluctuations, or masticatory forces, all of which play important roles in the oral environment. The absence of saliva eliminates potential effects such as pellicle formation and lubrication, which may influence abrasion behavior. Similarly, the lack of thermal cycling and pH variations does not reflect the dynamic chemical and thermal challenges present in the oral cavity, which may affect material degradation over time. In addition, masticatory forces were not simulated, although they may contribute to surface wear and fatigue under clinical conditions. Therefore, the brushing simulation represents only a limited aspect of intraoral conditions, and the results should be interpreted with caution when extrapolating to clinical practice. Brushing parameters were standardized and may not reflect individual oral hygiene habits. Only the Ra parameter was evaluated, and other surface topography parameters were not analyzed, while SEM observations were qualitative. In addition, dentifrice abrasivity was not directly measured, as parameters such as relative dentin abrasivity (RDA) values and abrasive particle characteristics were not evaluated. This limits the ability to attribute the observed differences among dentifrices specifically to their abrasive properties alone. Another limitation of this study is the use of only the Ra parameter for surface characterization. Although Ra is widely used and provides a general indication of surface roughness, it may not fully reflect the complexity of surface topography. Additional parameters, such as three-dimensional surface analyses, could provide a more comprehensive evaluation.

## Conclusions

Within the limitations of this study, surface roughness after simulated toothbrushing was significantly influenced by the interaction between restorative material and dentifrice. Additively manufactured resin-based restorative materials demonstrated material-dependent responses that were not consistently different from those of conventional composite resins under the tested brushing conditions.

## Supplementary Information

Below is the link to the electronic supplementary material.

Supplementary Material 1

Supplementary Material 2

Supplementary Material 3

Supplementary Material 4

Supplementary Material 5

Supplementary Material 6

Supplementary Material 7

Supplementary Material 8

Supplementary Material 9

Supplementary Material 10

Supplementary Material 11

Supplementary Material 12

Supplementary Material 13

Supplementary Material 14

Supplementary Material 15

Supplementary Material 16

Supplementary Material 17

Supplementary Material 18

Supplementary Material 19

Supplementary Material 20

Supplementary Material 21

Supplementary Material 22

Supplementary Material 23

Supplementary Material 24

Supplementary Material 25

Supplementary Material 26

Supplementary Material 27

Supplementary Material 28

Supplementary Material 29

Supplementary Material 30

## Data Availability

Data are available on request from the authors.
